# Progressive engineering of a homing endonuclease genome editing reagent for the murine X-linked immunodeficiency locus

**DOI:** 10.1093/nar/gku224

**Published:** 2014-03-25

**Authors:** Yupeng Wang, Iram F. Khan, Sandrine Boissel, Jordan Jarjour, Joseph Pangallo, Summer Thyme, David Baker, Andrew M. Scharenberg, David J. Rawlings

**Affiliations:** 1 Center for Immunity and Immunotherapies, Seattle Children's Research Institute, Seattle, WA 98101, USA; 2 Department of Biochemistry, University of Washington, Seattle, WA 98195, USA; 3 Pregenen Inc., Seattle, WA 98103, USA; 4 Departments of Pediatrics and Immunology, University of Washington, Seattle, WA 98195, USA

## Abstract

LAGLIDADG homing endonucleases (LHEs) are compact endonucleases with 20–22 bp recognition sites, and thus are ideal scaffolds for engineering site-specific DNA cleavage enzymes for genome editing applications. Here, we describe a general approach to LHE engineering that combines rational design with directed evolution, using a yeast surface display high-throughput cleavage selection. This approach was employed to alter the binding and cleavage specificity of the I-Anil LHE to recognize a mutation in the mouse Bruton tyrosine kinase (Btk) gene causative for mouse X-linked immunodeficiency (XID)—a model of human X-linked agammaglobulinemia (XLA). The required re-targeting of I-AniI involved progressive resculpting of the DNA contact interface to accommodate nine base differences from the native cleavage sequence. The enzyme emerging from the progressive engineering process was specific for the XID mutant allele versus the wild-type (WT) allele, and exhibited activity equivalent to WT I-AniI *in vitro* and *in cellulo* reporter assays. Fusion of the enzyme to a site-specific DNA binding domain of transcription activator-like effector (TALE) resulted in a further enhancement of gene editing efficiency. These results illustrate the potential of LHE enzymes as specific and efficient tools for therapeutic genome engineering.

## INTRODUCTION

Homing endonucleases (HEs) are sequence-specific enzymes that recognize and cleave DNA at long target sites (typically 20 bp). They are typically encoded within introns or inteins, and behave as mobile genetic elements that copy their genetic information into intron- or intein-less alleles of their host gene. This genetic mobility is catalyzed by HE endonuclease-mediated DNA double-strand breaks (DSBs) in intein/intron-less alleles of the host gene. This facilitates repair by homologous recombination using the intron- or intein-containing gene, resulting in copying of the intron or intein into the new allele site (1,2).

LAGLIDADG homing endonucleases (LHEs) are a particularly attractive system for the development of gene-specific reagents because they possess 20–22 bp recognition sites, and cleavage activity is tightly coupled to DNA target site recognition (1,3,4). A variety of approaches have been applied to generate LHE variants with new cleavage specificities, most of them involving ‘local’ variant library generation through random mutation or structural-based modification of the LHE protein interface that contacts the DNA target site, followed by selection based on DNA cleavage or recombination activities (5–8). These methods currently are able to generate variants with changes in cleavage specificity in a ‘local’ region of the LHE DNA/protein interface covering a relatively small number of contiguous base pairs. For physiologic targets, where multiple base pair mismatches must be targeted by variants that possess alterations in adjacent or overlapping regions of the DNA/protein interface, the engineering of LHE variants with high specificity and activity requires combinations of local changes that often include conflicting sets of amino acid (AA) changes in the interface. The development of methods to overcome limitations in large scale LHE re-specification is necessary to expand the application of LHEs to such extremely challenging targets.

Using a yeast surface display high-throughput cleavage selection system (9), here we show the application of rational design with directed evolution in a progressive approach to achieve a large scale re-engineering of the I-Anil homing endonuclease to specifically recognize a unique sequence in the mouse Bruton tyrosine kinase (*Btk*) gene differing by 9 bp from the native I-AniI sequence. Fusion of this enzyme to a sequence-specific TALE DNA binding domain was used to further increase the activity and specificity of the most refined variant for the XID target site. Taken together, our results provide a roadmap for engineering of LHEs to create highly specific and active reagents for therapeutic genome engineering.

## MATERIALS AND METHODS

### DNA constructs and substrates for binding and cleavage assays

The I-Anil scaffold used here is the Y2 variant, containing two additional mutations, F13Y and S111Y, which enhance both DNA-binding affinity and cleavage efficacy (10). The TALE repeat variable diresidue (RVD) arrays were assembled using Golden Gate TAL effector kit, and the TAL effectors were fused to the N-terminus of XID through a Zn4 linker (VGGS) (11,12). The 52 bp HE substrates were generated by PCR using single-strand oligonucleotides as template with HE recognition sites in the middle flanked by 16 bp primer binding site on each end. Biotin and fluorophore labels were introduced by 5′ biotin-conjugated primer and 3′ Alexa Fluor-647-conjugated primer, respectively. HE substrates were purified from single-stranded contaminants by Exo1 digestion (New England Biolabs) and size exclusion through a G-100 column (GE Healthcare), then analyzed for purity by gel electrophoresis (determined to be >98%) (9).

### Yeast growth, transformation, library construction and plasmid recovery

*Saccharomyces cerevisiae* strain EBY100 was transformed using the lithium-acetate (LiAc) method (13). For randomization library construction, randomization oligos with degenerative code on selected bases were ordered from Sigma. After PCR amplification, oligos were cloned into pETCON2 vector through homologous recombination in yeast. The distribution of codon frequencies was verified by sequencing an unselected library and determined to exhibit no major biasing of the type at positions of randomization (9). For random mutagenesis library construction, error-prone PCR was performed over selected region of the I-Anil variant using the GeneMorph-II Random Mutagenesis kit (Stratagene) according to the manufacturer's protocol. Library size was determined by plating serial dilutions on selective plates. Mutation distribution and frequencies were verified by sequencing an unselected library and determined to be in the range of 7–10 mutations per kilo base with no major biases in the type or position of mutations. Yeast propagation was performed in the presence of 2% raffinose + 0.1% glucose at 30°C for at least 12 h prior to induction. Cells were induced in 2% galactose for 2–3 h at 30°C followed by 18–26 h at 20°C. Plasmids were isolated from yeast populations using the Zymoprep-II kit (Zymo Research) and transformed into *Escherichia coli* DH5α by heat shock for amplification and/or sequencing. Sequencing was performed on 40–60 clones for a given selection output.

### Yeast surface cleavage and sorting

The yeast surface-based cleavage assay has been described previously (9). In brief, ∼30–100 × 10^6^ (at least 3-fold the size of the input population) induced cells were stained first with 1:300 dilution biotinylated anti-HA (Covance), then with pre-conjugated streptavidin-PE:biotin-DNA-A647 in a yeast binding buffer containing 180 mM KCl, 10 mM NaCl, 10 mM HEPES, 0.2% bovine serum albumin (BSA), 0.1% galactose, pH 7.5. Samples were then washed twice in the cleavage buffer containing 150 mM KCl, 10 mM NaCl, 10 mM HEPES, 10 mM K-glutamine, 0.5 mg/ml BSA, pH 8.25, and then transferred to cleavage buffer containing 7.5 mM of either CaCl_2_ or MgCl_2_, and placed at 37°C for the indicated time points. The reaction was stopped by transferring cells to three reaction volume cold staining buffer with 1:200 dilution FITC-conjugated anti-Myc antibody (LCL labs) for HE surface expression staining. The catalytic activity of HEs was measured by the decrease in Alex647 fluorescence intensity associated with dsOligo cleavage and release from yeast surface on a BD ARIAII cytometer, and resulting data were analyzed using Flowjo software (Tree Star). For XID-Ani libraries, ∼0.3–1% population with the highest cleavage activity were sorted for enrichment, and each library was enriched for three times before final analysis.

### *In vitro* cleavage assay and cleavage specificity

3 × 10^6^ displaying yeasts with an estimated concentration of 1–10 nM in a 40 μl reaction (assuming 10^4^–10^5^ molecules per yeast cell surface) (9,14) were incubated with cleavage buffer + 20 nM Alexa-647-conjugated dsOligo with 5 mM MgCl_2_, 5 mM dithiothreitol (DTT) and placed at 37°C for 1 h. The reaction was stopped by adding 50 mM ethylenediaminetetraacetic acid (EDTA) and DNA sample buffer. After spinning down, 20 μl of supernatant was loaded on a 10% non-denaturing polyacrylamide gel. HE cleavage sites are in the middle of oligo substrates, which will generate two products of the same size in cleavage assays. The cleavage product detected in *in vitro* cleavage assay is the 3′ half with Alexa Fluor-647 label. Quantification was performed with an Odyssey infrared imaging system (Li-Cor Biosciences), and cleavage activity was calculated by ratio of cleaved substrate to total substrate. The specificity profiles of XID-Ani and WT-Ani were generated by determining the *in vitro* cleavage of the enzyme to all 60 possible target sequences with one of the three other bases at each position. The percentage of cleavage was normalized to the cleavage of native target sequence.

### Yeast surface-based binding assay

The yeast surface-based binding assay and affinity calculation has been described previously (9). Briefly, 1 × 10^6^ displaying yeasts were incubated in 50 μl staining buffer containing biotin-labeled dsOligo ranging from 1 to 500 nM at 4°C for 2 h. After washing twice with excess staining buffer, yeasts were co-stained with streptavidin-PE (BD biosciences) and FITC-conjugated anti-Myc antibody for another 30 min. The binding affinity of HEs was measured by the median PE fluorescence value of around 10% selected population based on equal Myc epitope yeast surface expression on a BD LSRII cytometer, and resulting data were analyzed using Flowjo software. The median PE values were plotted versus dsOligo concentrations using the Levenberg–Marquardt (LM) algorithm in the VisualEnzymics (SoftZymics) module for IGOR Pro 6 (WaveMetrics).

### *In cellulo* assay in HEK293T cells and primary MEFs

I-Anil (tgaggaggtttctctgtaaa), XID (agtgcctgtttctcttgact), Ani-XID (agtgcctgtttctcttgactctgaggaggtttctctgtaaa) and TALE-XID (tcacctttaaacttcaagaagtgcctgtttctcttgact) target sites were inserted into Traffic Light Reporter (TLR) vector using standard molecular biology techniques, and corresponding lentivirus was produced as described previously (15,16). Reporter HEK293T cells were generated by transducing cells with serial-diluted reporter lentiviral vectors (LVs) to obtain a population of cells with single copy chromosomal integration, and selected by adding 1 μg/ml puromycin in the culture medium 48 h after transduction. The reporter cells were sorted against mCherry fluorescence to remove background resulting from integration errors. Open reading frames for WT-Ani, XID-Ani, and TALE-XID enzymes were amplified by PCR and ligated into the CVLlentiviral backbone, which also co-expresses blue fluorescent protein (BFP) by T2A peptide linker. Open reading frame for the 3′ repair exonuclease 2 (TREX2) was amplified by PCR and ligated into either CVL lentiviral or pEndo backbone, which also co-express iRFP by T2A peptide linker. For TLR assay, 1.5 × 10^5^ human embryonic kidney (HEK) reporter cells were seeded in a 12-well plate, and transiently co-transfected with 0.8 μg WT-Ani/XID-Ani/TALE-XID and TREX2 expression constructs using X-tremeGENE 9 DNA transfection reagent with manufacturer protocols (Roche Applied Science). Seventy-two hours after transfection, the cleavage activity of enzymes was measured by the percentage of mCherry positive cells, which represents double-strand break-induced mutagenic non-homologous end-joining (NHEJ) events, within a BFP marked nuclease-expressing population. Genomic DNA was isolated from BFP marked cells, and the precise cleavage rate of integrated target site was determined by sequencing after PCR amplification and cloning (CloneJET™ PCR Cloning Kit—Thermo Scientific). For homology-directed repair (HDR) assay, the 11RVD-TALE-XID nuclease was amplified by PCR and ligated into the CVL lentiviral backbone with d14GFP donor template. Seventy-two hours after transfection, HDR and NHEJ events were measured by the percentage of GFP and mCherry positive cells within a BFP positive population, respectively, and the precise gene modification rates within this population were determined by genomic sequencing. Mouse embryonic fibroblasts (MEFs) were isolated from homozygous XID embryos at 12–14 days of gestation. MEFs were cultured in Dulbecco's Modified Eagle's medium supplemented with 2 mM glutamine, 10 mM HEPES and 10% fetal bovine serum (FBS). For XID MEF experiments, 1.2 × 10^6^ cells were plated over 6 cm dishes. The following day, cells were transduced with LVs expressing 6RVD-TALE-XID and TREX2 in the presence of 4 μg/ml polybrene. Seventy two hours post-transduction, BFP and iRFP double positive cells were sorted and re-seeded in a 6 cm dish. Ten days after transduction, cells were harvested for genomic DNA. XID and its homologous sites were amplified from the harvested DNA and disruption rates were determined by genomic sequencing. All PCR primers used for genomic amplification were listed in Supplementary Table S3.

## RESULTS

Human XLA is a rare X-linked genetic disorder caused by mutations in the human *BTK* gene, which is expressed at all stages of B-lineage development and is required for pre-B cell expansion and mature B cell survival and activation (17,18). XLA patients lack mature B cells and immunoglobulin, and experience recurrent bacterial infections. Current life-long antibody replacement therapy is only partially effective, is expensive, and is associated with several long-term complications. While gene addition therapy with recombinant gammaretroviral and lentiviral vectors has shown promise (19,20), these approaches have the potential to cause insertional mutagenesis and gene expression mis-regulation. An ideal method for therapy of XLA would be to directly repair the BTK mutation in hematopoietic stem cells by double-strand break-induced homologous recombination (3). However, to achieve this efficiently requires the identification of a *Btk*-specific nuclease reagent with sufficient cleavage specificity for therapeutic use.

### Target selection and cluster-based engineering of an XID-specific variant of I-AniI

**Figure 1.**
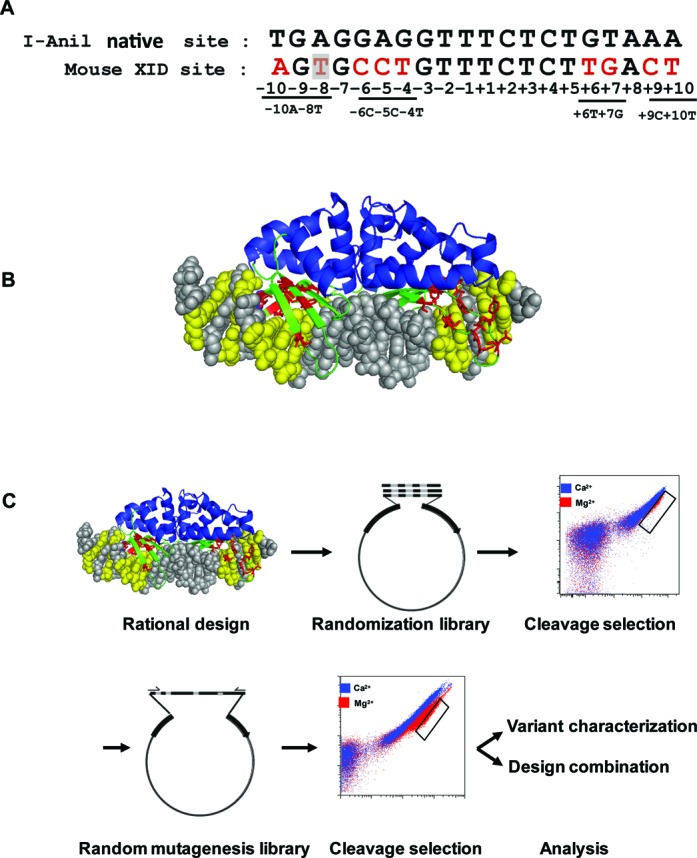
Target selection and cluster-based engineering of an XID-specific variant of I-AniI. (**A**) Alignment of the native I-Anil LHE recognition sequence with mouse XID site. Mismatches (red) were divided into four clusters. The single base XID mutation at −8 position (C→T) was shaded in gray. (**B**) DNA interface AA residues (red) targeted to DNA sequence mismatches (yellow) were selected for randomization. (**C**) The workflow for engineering I-Anil toward XID target site. Based on rational design, AA residues targeted to mismatch clusters were selected for randomization to generate yeast libraries. After cleavage selection, random mutations were introduced into selected active variants by error-prone PCR to generate random mutagenesis library for further cleavage selection. Finally, selected designs were characterized and combined to generate XID full site enzyme.

To explore the potential of using LHEs as gene modification tools for therapy of XLA, we selected a single base pair mutation within Exon 2 of the murine *Btk* gene as target site (this mutation is found in the XID mouse, a murine model of human XLA) (19,20). We endeavored to re-program the specificity of the LHE, I-Anil, to uniquely target the mutant allele. This was a significant undertaking, as there are 9 bp differences between wild-type (WT) I-Anil target sequence and XID sequence (Figure 1A), including multiple residues known to be extremely important for I-AniI activity. Furthermore, targeting this site required engineering of AAs that comprise the protein–DNA interface contacting both the −7 to −5 and the +5 to +7 positions of the target site, residues that previous combinatorial strategies elected to bypass due to the high number base pair contacts in these positions (21).

Recently, a number of I-AniI variants have been isolated that cleave target sites with single base pair mismatches from the original I-AniI target (22). As the direct combination of re-designed variants targeted to single base mismatch did not generate active enzymes (−6C−5C−4T and +6T+7G) (data not shown), we selected a ‘cluster’-based engineering strategy by dividing the XID sequence into four ‘cluster’ of mismatched residues (−10A−8T, −6C−5C−4T, +6T+7G and +9C+10T) based on enzyme structure and Ani/XID target sequence mismatch positions (Figure 1A and B). Based on structural information, AA residues interacting with these clusters were selected for randomization. I-AniI variants bearing alterations in these residues were incorporated into a yeast surface expression vector, thereby taking advantage of the high homologous recombination efficiency in yeast to generate an LHE yeast surface display library. Using a previously reported yeast surface cleavage assay (9), the yeast library was subjected to three rounds of flow cytometry-based selection to enrich highly active variants. To further increase enzyme activity, random mutations were introduced by error prone PCR into open reading frames (ORFs) of variants emerging from primary screens targeting each cluster. After three rounds of cleavage selection from the random mutation library, enzyme-expressing DNA vectors were isolated from the final enriched populations, and individual clones were sequenced and characterized *in vitro* (Figure 1C; with details of engineering experiments performed for each cluster provided in Supplementary Figures S1–S4). As shown in +6T+7G and −6C−5C−4T cluster libraries (Supplementary Figure S2D, S3F, and Supplementary Table S1), variants with the highest cleavage activity were highly enriched in the final selected population (up to 80% for a single variant), and these clones typically exhibited similar patterns of AA variation at the diversified positions.

### Combination of cluster designs into an enzyme able to cleave the XID target site

Using the output of the cluster selections, we generated libraries in which cluster designs were combined to create enzymes with active plus (+) and negative (−) half sites. However, we found that directly combining active (+) and (−) half-site designs from these libraries did not generate active full site variants, suggesting conformational conflict between (+) and (−) half-site designs (data not shown). Therefore, we evaluated partial half-site combinations, and were able to isolate a −6C−5C−4T+6T+7G+9C+10A-Ani variant with moderate activity on yeast surface. Using this ORF as template for a random mutagenesis library (Supplementary Table S1, Supplementary Figure S4A), we screened for variants that exhibited increased activity using our flow cytometry-based cleavage assay. From this screen, we identified a R243W mutation that was highly enriched in the most active population (Supplementary Figure S4B and C). Structural analysis of the R243 residue indicated that it is positioned a short distance from the native +9 A:T pair, and mutation at that position to C:G pair causes steric clash between this residue and +9 nucleotide pair. When combining (+) and (−) site designs, we speculate that this steric clash affects the positioning of catalytic domain, an effect that can be compensated by the R243W mutation. This conclusion is further supported by the ability of the R243W mutation to rescue all previously inactive XID variants, following which their cleavage activity could be further increased by random mutagenesis and selection (Supplementary Table S1, Supplementary Figure S4D and E).

### *In vitro* characterization of XID-Ani

**Figure 2.**
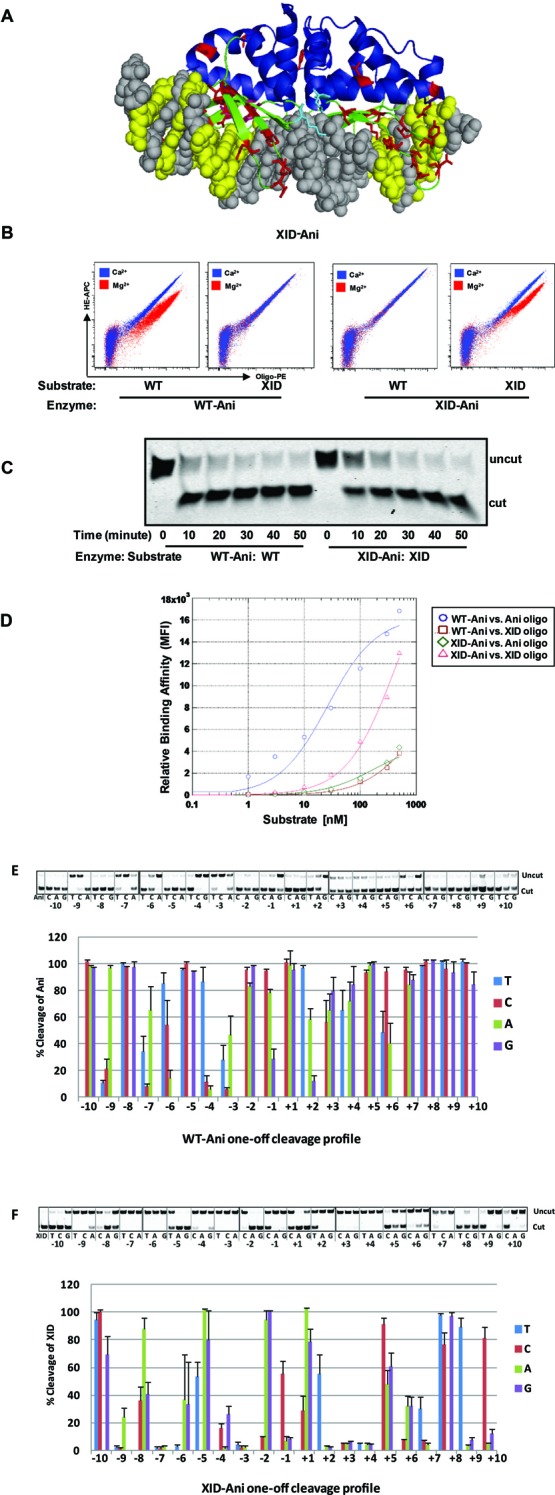
*In vitro* characterization of XID-Ani enzyme. (**A**) The final selected XID-Ani variant has 31 AA mutations (red) from WT-Ani. (**B**) XID-Ani showed similar cleavage efficacy as WT-Ani on yeast toward its target sequence as indicated by allophycocyanin (APC) signal shift in the presence of Mg^2+^. In contrast, XID-Ani exhibits no activity toward the WT-Ani sequence. (**C**) XID-Ani demonstrated similar cleavage *in vitro* kinetics toward its target site as WT-Ani. Top bands show uncut dsOligo candidate HE substrates. Lower bands represent the 3′ half of HE-cleaved dsOligo substrates detected on the basis of the Alexa Fluor-647 label. (**D**) XID-Ani showed lower binding affinity than WT-Ani toward their respective target sites on a yeast surface-based binding affinity titration assay. (**E** and **F**) One-off *in vitro* cleavage profiles for WT and engineered XID-Ani. Upper panels show cleavage activity of WT-Ani and XID-Ani toward their respective targets and one-off sites in an *in vitro* cleavage assay. Lower panels show quantification of relative cleavage efficacy with cleavage toward Ani and XID sites set as 100%, respectively. Quantification and standard error were calculated from three independent experiments.

The final selected XID-Ani variant (enriched up to 80% in the final population) includes 31 AA alterations from WT-Ani, with nearly half of them (14) selected from random mutagenesis libraries (Supplementary Table S1, Figure 2A). In a yeast surface-based cleavage assay, XID-Ani showed similar cleavage efficacy as WT-Ani without detectable activity toward the WT target (Figure 2B). Once dissociated from the yeast surface, XID-Ani also showed similar cleavage kinetics as WT-Ani in an *in vitro* cleavage assay (Figure 2C). We previously showed that the specificity of re-designed enzymes targeted to partial XID site (−6C, +6T+7G) has significantly improved specificity compared with WT-Ani (Supplementary Figures S2D and S3B). To evaluate the specificity of the engineered XID-Ani enzyme, we compared one-off cleavage specificity profile of XID-Ani with WT-Ani, and also measured their binding affinity (Figure 2D). ‘One-off’ target site specificity for XID-Ani ranged from relatively high at 9 bp positions (where the efficiency of cleavage of any other three bases was less than 50% of the XID target base), to partial or complete degeneracy at 11 positions (where at least one other base was cleaved with an efficiency >50% of the XID target site) (Figure 2F). This is a significant improvement from the WT-Ani, which showed partial or complete degeneracy at 19 positions (Figure 2E) (9), and is explained at least in part by the fact that the re-designed enzyme has a reduced affinity for the XID target site—thus, any single base pair mismatch is more likely to compromise binding to a sufficient extent that enzymatic activity is compromised. The improved specificity of XID-Ani using the highly sensitive flow cytometry assay is an important achievement, as it emphasizes the capacity of the I-AniI scaffold to be engineered so as to achieve a high efficiency of on-site cleavage while reducing off-target cleavage over nearly the entire DNA/protein interface, an important consideration for therapeutic applications where specificity is paramount.

### *In cellulo* performance of XID-Ani

**Figure 3.**
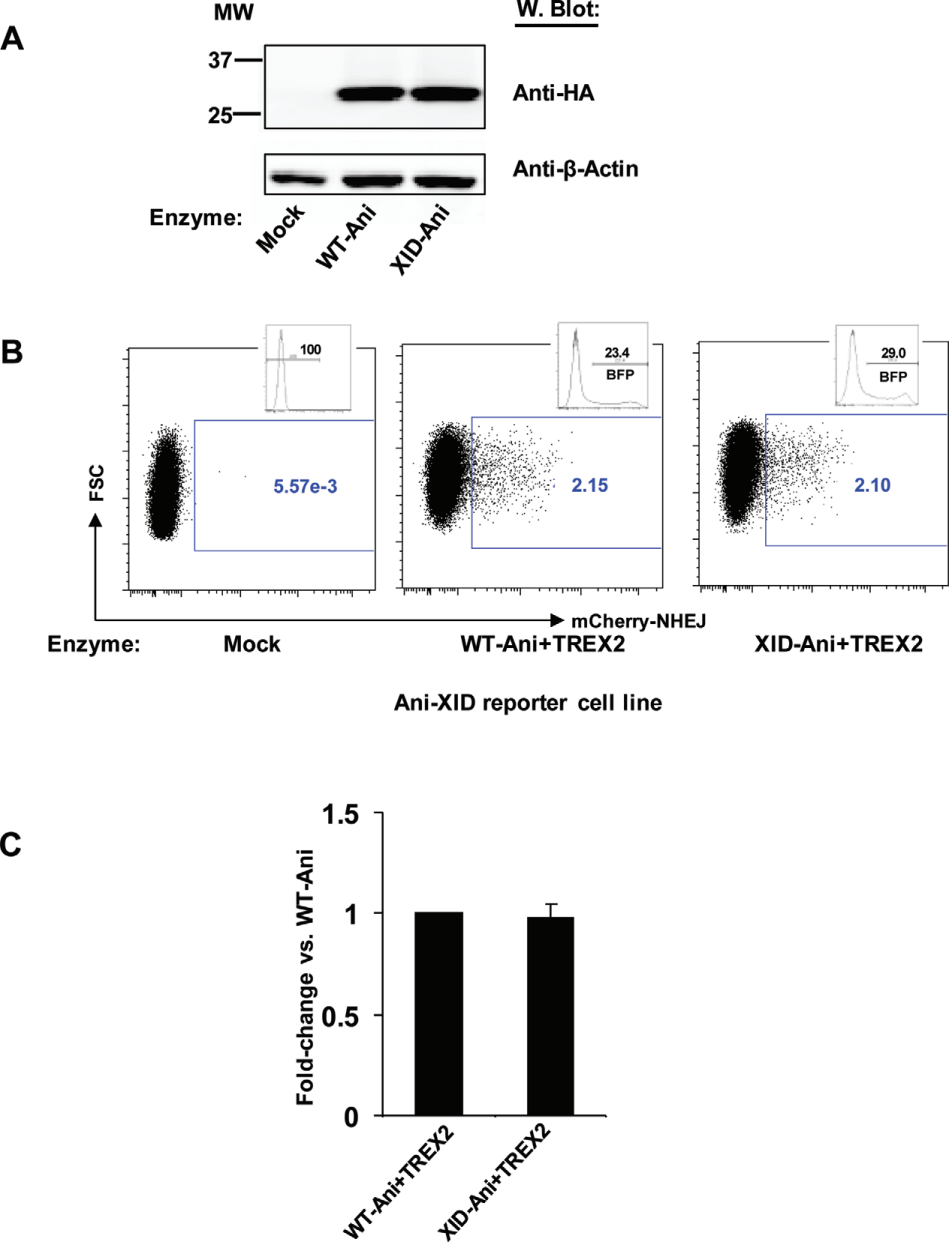
*In cellulo* performance of XID-Ani. (**A**) Equivalent expression levels of WT-Ani and XID-Ani in HEK293T cells. Cells were co-transfected with vectors expressing WT-Ani or XID-Ani and a vector expressing TREX2 with a *cis*-linked BFP reporter. Seventy two hours later, protein expression was detected by western blot using an anti-HA antibody. β-Actin was used as loading control. (**B**) When co-expressed with TREX2, WT-Ani and XID-Ani induced a similar level of NHEJ events in 293T reporter cells containing WT-Ani and XID target sequences. The cleavage activity was measured as percentage of mCherry positive events within BFP expressing cells (or within the total cell population in mock-treated cells). Histogram showing gating of non-transfected (mock) or BFP expressing cells is displayed in upper right quadrant of each FACS plot. (**C**) Quantification of NHEJ activity was calculated from three independent experiments.

To characterize the activity of the re-designed XID-Ani in a cell-based model, we used a previously described TLR system, that is able to report the capacity of an enzyme to generate both mutagenic NHEJ and targeted HDR (16). The *in cellulo* cleavage activity of XID-Ani and WT-Ani were measured by monitoring each enzyme's ability to generate mCherry positive cells when co-expressed with the 3′ exonuclease TREX2 (TREX2 degrades the 3′ overhang of DSBs generated by HEs, and thus its overexpression leads to increase rates of end processing, and so an increased probability that a cleavage event will be converted to a mutagenic outcome (23)). To facilitate direct *in cellulo* cleavage efficacy comparison, a reporter cell line in which both XID and Ani target sites were included in a single TLR was utilized for both enzymes. Reporter cell lines with single target sites were used, in parallel, as controls. Both enzymes were stably expressed in HEK293T cells with similar levels of protein detected by western blot (Figure 3A). Similar to WT-Ani, XID-Ani only induced NHEJ events in 293T reporter cells that possessed the Ani-XID target, but not the WT-Ani target site alone (Figure 3B, Supplementary Figure S5). The cleavage efficacy of XID-Ani *in cellulo* was similar to WT-Ani, consistent with their observed relative *in vitro* cleavage activities (Figure 3B and C).

### TALE DNA binding domain fusion with XID-Ani significantly increases its cleavage efficacy

**Figure 4.**
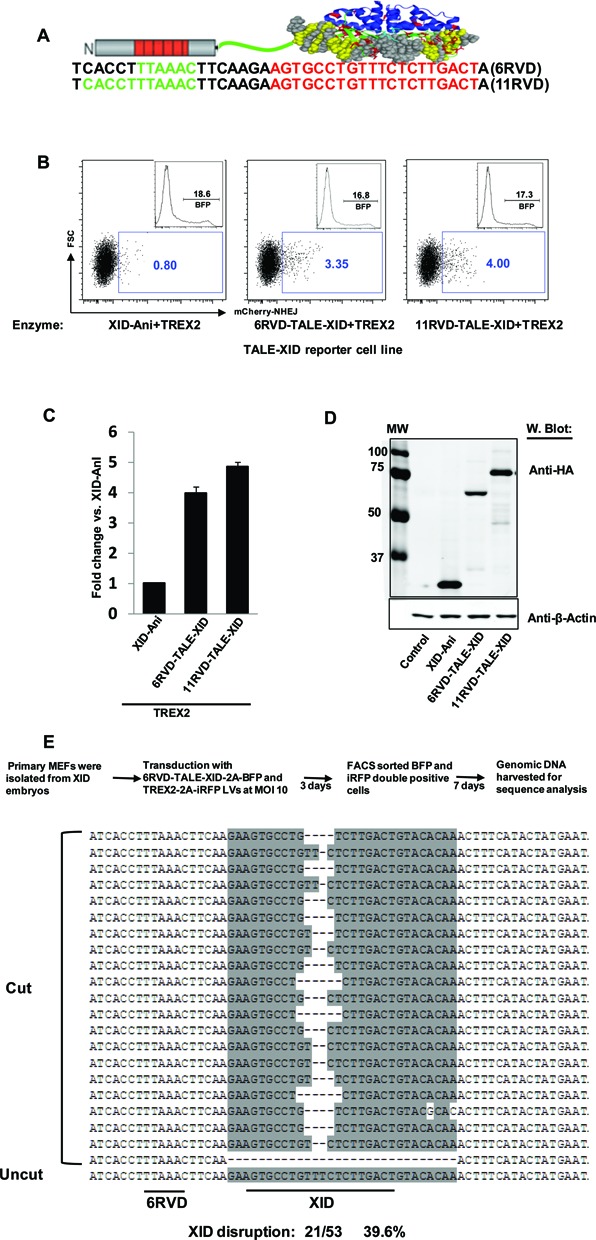
Fusion of TALE DNA binding domain with XID-Ani significantly increases its cleavage efficacy. (**A**) Schematic illustration of TALE-XID enzymes (6RVD and 11RVD) and their recognition sequences in mouse XID native locus (green: TALE binding site; red: XID site). (**B**) TALE-XID enzymes with 6RVDs or 11RVDs significantly increased cleavage efficacy in HEK293T TLR cells containing the native XID locus sequence when co-expressed with TREX2. Reporter cells were co-transfected with vectors expressing TALE-XID enzymes and a vector expressing TREX2 with a *cis*-linked BFP reporter and cleavage activity was measured as percentage of mCherry positive events within BFP expressing cells. Histogram showing BFP expression is displayed in upper right quadrant of each FACS plot. (**C**) Quantification of data was calculated from three independent experiments. The cleavage activity of XID-Ani based on percentage of mCherry was set as one (**D**) XID-Ani, 6RVD-TALE-XID and 11RVD-TALE-XID were stably expressed in HEK293T cells without degradation as detected by western blot analysis using an anti-HA antibody. β-Actin was used as loading control. (**E**) In the presence of TREX2, transduction using a LV expressing codon diverged 6RVD-TALE-XID disrupts ∼40% of endogenous XID sites in primary XID MEFs. Upper panel shows schematic of experimental approach and lower panel displays genomic sequencing data from disrupted loci.

To further increase the efficiency and specificity of the XID-Ani enzyme, we used a recently developed TALE–LHE fusion hereafter referred to as megaTAL architecture (12). We created megaTAL fusion enzymes with TALE DNA binding domains targeted to 6 or 11 bp of the native *Btk* locus sequences located upstream of murine XID site in conjunction with a 7 bp spacer (Figure 4A). The cleavage efficacy of these TALE-XID endonucleases was compared with XID-Ani LHE in a HEK293T TLR cell line with integrated TALE-XID sequence (Figure 4A). With both TALE array fusions, the ‘on-site’ cleavage efficacy of TALE-XID was substantially increased as reported by the TLR flow cytometry readout (Figure 4B and C; with level of protein expression demonstrated in Figure 4D). As cleavage measured by TLR cell lines has been found to under report true mutation rates in some cases (24), we also assessed cleavage at the XID target in this line via amplicon-based sequencing. Analysis of amplicon sequences demonstrated that nearly complete disruption of the XID site was achieved in cells co-expressing 11RVD-TALE-XID and TREX2 (27/28 readouts, 96.4%). Importantly, the ratio of cleavage efficacy determined by genomic sequencing between different enzymes was consistent with the ratio indicated by the mCherry reporter readout of the TLR, validating the use of the TLR as a tool for relative comparisons of enzymatic activity (data not shown).

Because we were not able to compare relative binding affinity of 11RVD versus XID-Ani using the yeast surface-based binding assay (due to the difficulty of expressing the TALE domain on the yeast surface), we compared the relative contribution of TALE and HE by combining different TALE domains and HE variants. Using a 17 RVD TALE (‘L538’—TCATTACACCTGCAGCT) (25), we were able to increase the cleavage activity of XID-Ani. We also obtained similar cleavage rates using another XID variant with nearly identical turnover rate (*K*_cat_) but a significantly lower binding affinity and cleavage activity (∼20% of XID-Ani in the TLR assay). Based on these findings, we argue that the binding affinity of this 17 RVD TALE is sufficient to bring HEs with different binding affinity to their maximum activity. With the 11 RVD TALE (which has lower binding affinity than ‘L538’), the cleavage efficacy of this XID variant was around 70% of XID-Ani (data not shown). Based on this observation, we estimate the binding affinity of 11 RVD contributes 70–80% of the total observed activity, which is consistent with the data showed in Figure 4C.

We next determined the cleavage efficacy of the 6RVD-TALE-XID enzyme at the XID mutant *Btk* genomic locus. Primary MEFs derived from XID embryos were co-transduced by LVs expressing the 6RVD-TALE-XID and TREX2 (multiplicity of infection (MOI) of 10). Of note, the codons of the highly repetitive RVD array sequences were diverged to reduce sequence rearrangements occurring during reverse transcription (26) thereby permitting efficient LV packaging and expression of this novel nuclease without evidence for protein degradation (data not shown). Ten days after transduction, native XID target disruption rate was determined by genomic sequencing within cells marked by both viruses. Although the XID site is thought to be within the silent *Btk* locus in primary MEFs, nearly 40% disruption (21/53 readouts, 39.6%) was detected in XID MEFs, primarily including small deletions within the central four bases of XID-Ani enzyme recognition site (Figure 4E).

### Off-target cleavage of TALE-XID enzyme

One of the most important considerations for therapeutic application of rare-cutting endonucleases is maintaining a rate of off-target cleavage that is as low as possible (27). Although XID-Ani showed significantly improved specificity from WT-Ani, cleavage profiling identified 19 one-off cleavage sites tolerated by XID-Ani *in vitro* (based on >50% predicted cleavage activity at candidate target nucleotides; Figure 2F). To examine the specificity of TALE-XID when expressed *in vivo*, we searched for potential cleavage sites identified in the mouse genome. We identified 23 potential sites including: two sites with a 1 bp mismatch and 21 sites with 2 bp mismatches. Sequence and chromosomal positions of these sites are provided in Supplementary Table S2. The one-off cleavage profile predicted that the 1 bp mismatch sites (including the WT *btk* sequence) would be partially tolerated. Among the 2 bp mismatches, the cleavage profile predicted that seven sites would be tolerated (both single mismatches tolerated by XID-Ani) and 14 sites would not be tolerated (at least one mismatch is not tolerated).

**Figure 5.**
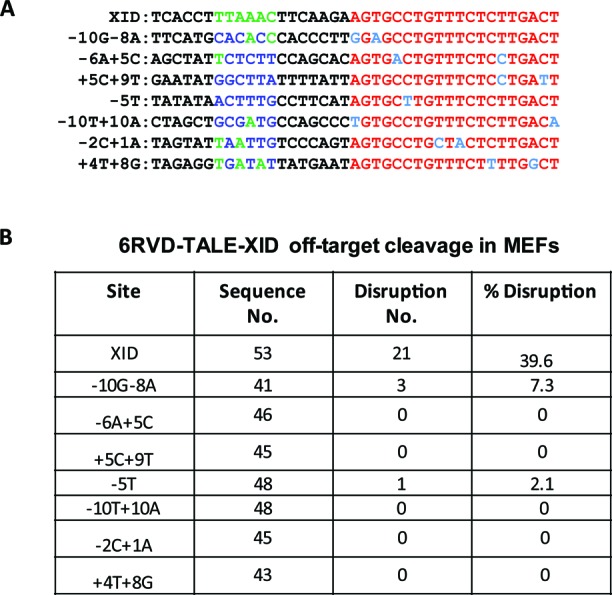
Assessment of potential off-target cleavage activity of TALE-XID enzyme. (**A**) Alignment of seven selected potential XID homologous sites in mouse genome. TALE, XID binding sites and mismatch positions are marked in green, red and blue, respectively. (**B**) Table showing 6RVD-TALE-XID off-target cleavage activity in XID MEFs. Potential sites were sequenced following PCR amplification of genomic DNA derived from XID MEF in which 40% XID site disruption had been achieved by LV TALE-XID expression (as in Figure 4E).

To directly test the rate of cleavage at predicted off-target sites *in vivo*, seven potential sites including candidates from each of these three categories were sequenced from XID MEF genomic DNA isolated from a cell population in which a 40% XID site disruption had been achieved by LV TALE-XID expression. As shown in Figure 5A, none of these sites possessed upstream homologous TALE binding sequences. No disruption was found at sites that were predicted *in vitro* to be cleavage resistant (−10T+10A, −2C+1A, +4T+8G). As for sites for which *in vitro* cleavage was observed, we detected no disruption at the −6A+5C and +5C+9T sites, but 7.3% (3/41 readouts) disruption at the −10G−8A site (Figure 5B). The tolerance of the −10G-8A site is likely due to their positions on nearby residues −10 and −8 within the N terminal loop, where WT I-AniI has little specificity, while the −6A+5C and +5C+9T are targeted by distinct domains of XID-Ani and, thus, are more likely to be incompatible with cleavage when combined together. Finally, we observed low-level, 2.1% cleavage activity at the partially tolerated −5T site. Importantly, the disruption rate for −10G−8A and −5T sites was at least 5-fold lower than that of the XID site, consistent with the observed difference in cleavage efficacy of XID-Ani with and without the fused TALE DNA binding domain in the TLR assay (Figure 4C). Considering the stringency of the assay, which enhances detection of nuclease-mediated disruption via extended lentiviral vector driven nuclease and TREX2 co-expression, these combined results further illustrate the importance of the TALE domain in specifically enhancing ‘on site’ activity.

### Catalysis of homologous recombination by the TALE-XID enzyme

**Figure 6.**
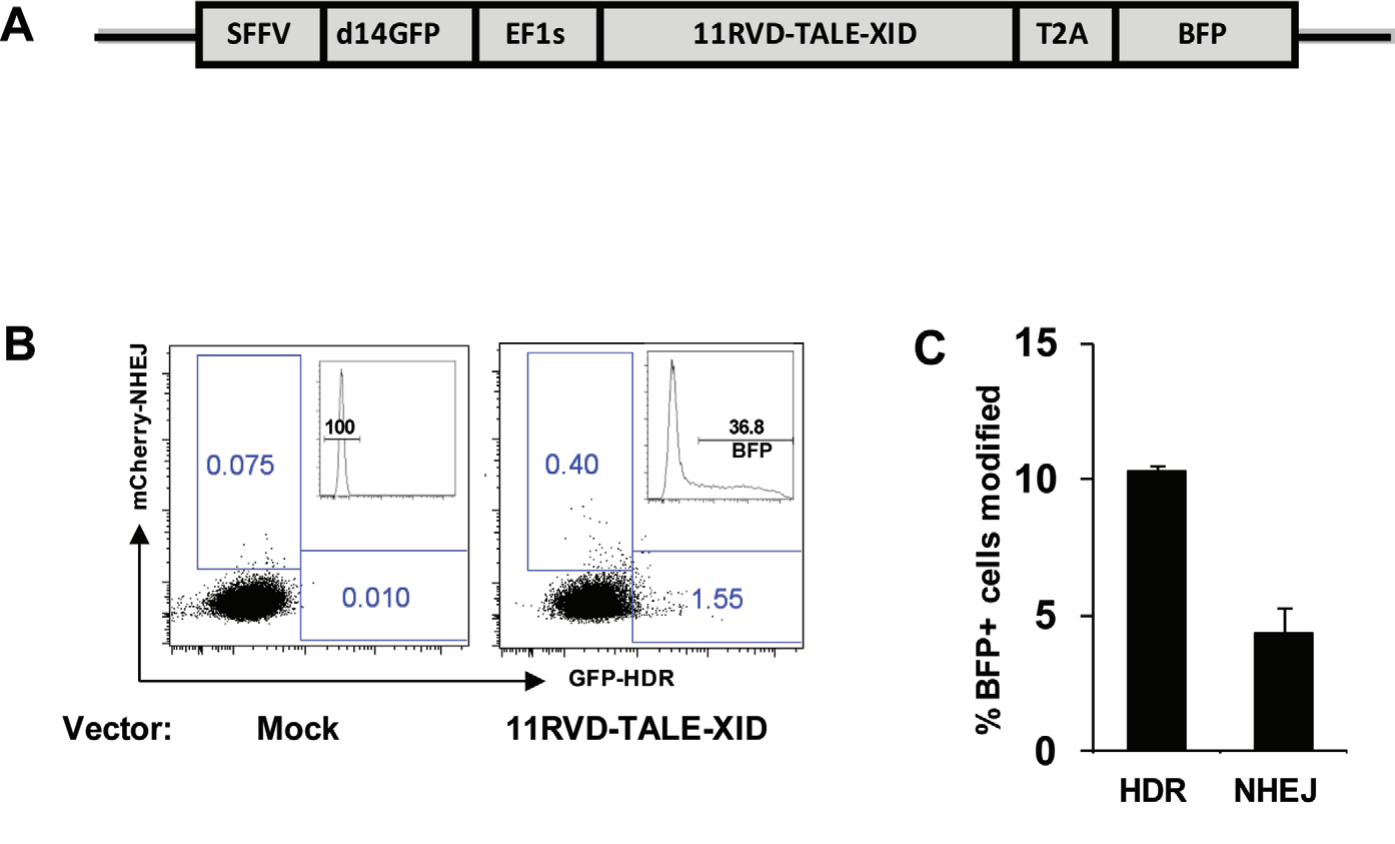
Catalysis of homologous recombination by the TALE-XID enzyme. (**A**) Schematic of vector construct expressing GFP donor template, TALE-XID enzyme, and *cis*-linked BFP reporter. (**B**) In the presence of donor template, 11RVD-TALE-XID induced both HDR and NHEJ in TLR cells containing the native XID locus sequence as shown by GFP and mCherry positive population, respectively. Reporter cells were transfected and HDR and NHEJ activity was measured within BFP expressing cells. Histogram showing BFP expression is displayed in upper right quadrant of each FACS plot. (**C**) The percentage of BFP positive cells modified by either HDR or NHEJ in TLR assay was determined by genomic sequencing. Data shown is the average of two independent experiments.

As the therapeutic purpose of the TALE-XID enzyme is gene repair, rather than gene disruption, we evaluated the use of the TALE-XID enzyme for catalysis of HDR. For this purpose, TLR cells were transfected with an expression vector containing both the 11RVD-TALE-XID and a d14GFP donor template (pRRL SFFV d14GFP EF1s 11RVD-TALE-XID T2A mTagBFP) in the absence of TREX2 (Figure 6A). Three days after transfection, NHEJ and HDR rates were read out based upon mCherry versus GFP positive cells, respectively. As shown in Figure 6B, a clearly detectable GFP+ population was detected, indicative of HDR. Similar to the NHEJ read out in the TLR assay, the percentage of GFP positive cells can underestimate the true HDR rate due to promoter silencing (24). Using a pair of primers specific for the inserted TLR target site, we amplified the reporter target and sequence analysis revealed that 10.3% of the reporter XID target sites were modified by HDR and 4.3% by NHEJ (Figure 6C). This high ratio of HDR to NHEJ highlights an important advantage of using a homing endonuclease-based reagent for gene editing—the ratio of HDR to NHEJ is typically substantially higher, likely due to differential processing of 3′ and 5′ breaks (24).

## DISCUSSION

Here, we present the results of a progressive re-design strategy applied to re-specify the cleavage site of an I-Anil LAGLIDADG homing endonuclease to a unique mutation site in the murine *Btk* gene. Using yeast surface display high-throughput cleavage selection, we were able to overcome neighbor effects in the highly integrated LHE interface by progressively assembling individual ‘half site’ enzymes followed by assembly of the engineered ‘half site’ into an active enzyme. Overall, our engineering effort achieved a 9 bp alteration of WT-AniI from its native sequence, and yielded an enzyme that exhibits significant specificity for the XID versus WT allele.

The cluster-based engineering approach used in this study involved randomization of DNA interface residues, and the initial variants isolated from such libraries were noted to nearly uniformly have significantly lower cleavage activity than the WT enzyme. Based on these observations, we infer that simultaneous changes of multiple protein sequences at the DNA interface region results in structural or conformational shifts that compromise enzyme activity. However, such changes have not been reliably predictable using current modeling techniques, making rational designs for compensation very difficult. In this study, we used a strategy of random mutagenesis following isolation of interface variants to identify those residues capable of improving enzyme activity. Within our final XID-Ani enzyme, 14 of 31 mutations were introduced by random mutagenesis. Of these, we speculate that E86D, F91L, D122N and C150S may increase XID-Ani solubility and stability on yeast surface. Notably, three of these residue alterations are naturally observed in a recently identified, highly active, I-Anil homolog, I-HjeI LHE (28). K39R, E63K, M66T, N226Y and R243W appear to be critical for enzyme activity, as they appear to have occurred in order to compensate for significant structural shifts caused by other mutations. The remaining five mutations (I55T, I64T, H76Q, R172K and K232E) may slightly affect enzyme activity through unclear mechanisms. Importantly, we were consistently able to apply random mutagenesis to identify variants with significantly increased cleavage activity, if the starting point was an enzyme with modest cleavage activity. However, we have not been successful in recovering active enzymes from randomly mutagenized non-active variants. We speculate that recovering activity from an inactive variant likely requires simultaneous changes in multiple residues, and thus results in a combinatorial problem whose complexity is beyond our present selection methods.

Our work emphasizes the difficulty that protein engineers are likely to encounter when attempting to combine AA changes individually selected to allow for altered specificity in adjacent or nearby areas of the LHE protein/DNA interface. Our first attempt to directly combine designs that produced single base-pair shifts in cleavage specificity failed to generate active variants, an observation which we attribute to neighbor effects between designs due to the highly integrated LHE protein/DNA interface. We also observed in several cases that active variants selected against a cluster (e.g. +6T+7G) were able to cut combined +6T and +7G but not +6T or +7G individually (Supplementary Figure S2D). This is consistent with structure-based predictions that these 2 bp are targeted by an overlapping and structurally dependent area of the protein/DNA interface, and thus separate designs would likely generate steric clashes that compromise enzyme activity. Similarly, our attempts to combine two adjacent regions targeted to −10A−8T and −6C−5C−4T by a direct combination strategy also did not work. This observation could be explained if the structural shift caused by incorporations of the −6C−5C−4T design had a neighbor effect on the loop region targeted to −10A−8T. Only after that shift was compensated by mutations from another region of the interface, was it possible to re-design this loop (Supplementary Figure S3G-I). Finally, the combination of XID (+) and (−) half designs, which reside in the I-Anil N-term and C-term domains, respectively, and target to distant sequences, unexpectedly did not generate any active variants as a direct combination. However, following random mutagenesis, a single R243W mutation was recovered that allowed all inactive variants to regain cleavage activity. We hypothesize that the re-designed half enzyme resulted in a shift in the catalytic residues, which was subsequently compensated for by the R243W mutation.

While we achieved significant success in resculpting a DNA/protein interface that allowed high specificity and activity toward the XID target, this interface was not able to achieve a high binding affinity for the XID site. Despite its reduced binding affinity *in vitro*, XID-Ani exhibited a level of activity in *in vitro* and *in cellulo* reporter assays that was equivalent to WT I-AniI. It also exhibited significantly improved specificity, critical for reducing the risk of off-target cleavage in therapeutic applications. We attribute the reduced binding affinity to the nature of the resculpted interface of the (−) half site, which disrupts several hydrogen bonds that are involved in generating the high affinity of WT I-AniI for its target site. We speculate that to achieve the same level of cleavage activity as WT I-AniI, the reduced binding affinity was partially compensated by an increased turnover rate (*K*_cat_).

Importantly, the introduction of a site-specific TALE DNA binding domain using the megaTAL platform was able to overcome the reduced binding affinity of XID-Ani for the XID site, thereby providing markedly improved *in vivo* activity. Fusion of XID-Ani to TALE DNA binding domains containing as few as six RVDs, resulted in markedly enhanced cleavage efficacy in both reporter cell lines and primary XID MEFs. The TALE DNA binding domain significantly increased XID-Ani cleavage efficacy to its target site but is not anticipated to alter the binding affinity of the XID-Ani cleavage head to its homologous sequence. Thus, we do not anticipate that the megaTAL fusion changes the intrinsic specificity of the HE cleavage head. However, by increasing on-site activity, a desired level of on-site modification can be achieved with a reduced protein expression level and/or a more limited period of expression thereby resulting in reduced off site cleavage. Thus, megaTAL fusions result in an increase in the effective specificity of the enzyme (12). We speculate that future LHE engineering for therapeutic applications may focus on the intentional development of low affinity, high specificity LHE's, with the planned addition of a TALE or other affinity enhancing domain to provide for site specific activity to a desired single target site.

Finally, a notable aspect of the performance of the XID-Ani megaTAL was that it was able to achieve a high ratio of HDR to NHEJ. High HDR to NHEJ ratios have typically been observed with homing endonucleases, whereas both zinc finger nucleases (ZFNs) and TALENs have typically yielded the reverse—rates of NHEJ that are higher than HDR (24). This is an important observation, as it suggests that the DNA ends created by megaTAL reagents are processed in a manner akin to their homing endonuclease cleavage heads, as opposed to being processed equivalently to a FOK-I based TALEN. It also supports the concept that gene editing reagents based on homing endonuclease cleavage domains may be superior choices for gene editing applications that are dependent on homology-directed repair.

In summary, here we have shown that a yeast surface display-based high throughput selection system for HE engineering can be applied in a progressive re-design strategy to execute an aggressive resculpting of an LHE protein/DNA interface. By adopting a progressive strategy that incorporated random mutagenesis to boost activity between interface resculpting steps, we were able to achieve a 9 bp alteration in cleavage specificity from the native target site. Furthermore, we show that compensating XID binding affinity through TALE DNA binding domains significantly improved the cleavage activity of the final enzyme, yielding a highly active and specific gene editing reagent able to catalyze high rates of homology-directed repair at its target site. The success of our progressive strategy in achieving a significant specificity shift and successful incorporation of the re-designed XID-Ani into the megaTAL format offer a roadmap for future LHE engineering projects aimed at creating highly specific and active gene editing reagents.

## SUPPLEMENTARY DATA

Supplementary Data are available at NAR Online.

SUPPLEMENTARY DATA
