# Supplementary material for: Progressive engineering of a homing endonuclease genome editing reagent for the murine X-linked immunodeficiency locus
Source: Nucleic Acids Res. 2014 Mar 25;42(10):6463–75. doi: 10.1093/nar/gku224 (PMC4041414; doi:10.1093/nar/gku224)
Supplement: SUPPLEMENTARY DATA [file supp_42_10_6463__index.html]

Progressive engineering of a homing endonuclease genome editing reagent for the murine X-linked immunodeficiency locus — SUPPLEMENTARY DATA 

# Progressive engineering of a homing endonuclease genome editing reagent for the murine X-linked immunodeficiency locus

## SUPPLEMENTARY DATA

**Files in this Data Supplement:**

- SUPPLEMENTARY DATA
