# Supplementary material for: Progressive engineering of a homing endonuclease genome editing reagent for the murine X-linked immunodeficiency locus
Source: Nucleic Acids Res. 2014 Mar 25;42(10):6463–75. doi: 10.1093/nar/gku224 (PMC4041414; doi:10.1093/nar/gku224)
Supplement: SUPPLEMENTARY DATA [file supp_gku224_nar-03352-f-2013-File009.pdf]

**SUPPLEMENTARY DATA**

To accompany Wang et al.

**TABLE AND FIGURE LEGENDS**

**Supplementary Figure S1. WT-Ani tolerates mismatches at -10A-8T and +9C+10T clusters.** Equal amounts of WT-Ani enzyme were incubated with 20 nM alternative Alexa-647-conjugated ds-oligo substrates as indicated at 37°C for 1 hour. Oligos were separated by 10% non-denaturing polyacrylamide gel and quantified with an Odyssey infrared imaging system (Li-Cor Biosciences). Due to tolerance of these two clusters, XID enzyme engineering was initiated with cluster +6T+7G and -6C-5C-4T.

**Supplementary Figure S2. XID (+) half-site enzyme engineering.** A. Based upon structural predictions, six AA residues (Y154, I164, S166, T189, K202 and T204) targeted to +6T+7G cluster were selected for randomization. To keep the theoretical library diversity within the range of yeast library size, randomization of T189 was limited to T, P, or R based on computational model prediction and previous selection. The remaining five residues were completely randomized with code NNK. The predicted diversity of such design is around $12.8 \times 10^6$, which was well represented by the corresponding yeast library with the size of 30 million. B. Data showing three rounds of cleavage selection of +6T+7G randomization library. For each round of selection, around 0.3% potential active variants from reaction with $Mg^{2+}$ were selected for continued culture. No obvious active population was observed until Round 3 selection (see small number of red events appearing just below the major population of APC/PE double positive events in blue). C. Cleavage activity of +6T+7G-Ani variants selected from randomization library (lib) was significantly improved after random mutagenesis of this library followed by selection. D. Cleavage activity and specificity of five highly enriched variants from mutagenesis library. Variants exhibited similar mutations on DNA interface residues (Supplementary Table S1), and most of them (V2 to V5) only cut +6T+7G cluster, but not +6T or +7G single mismatch. E. Using five +6T+7G-Ani variants as seed template, four residues (L156, N157, D160 and Y162) targeted to +9C+10T cluster were randomized to generate a XID (+) half-site library. F. Cleavage activity and specificity of (+) half-site active variants selected from randomization library on yeast surface and *in vitro*. G. XID(+)-Ani variant 1 ((+) V1) not only had the WT enzyme comparable cleavage activity *in vitro* and on yeast surface, but showed significantly improved specificity at the +9+10 cluster, thus was selected as template to assemble XID full site enzyme(Supplementary Table S1).

**Supplementary Figure S3. XID (-) half-site enzyme engineering.** A. Schematic of Ani design (T22S, E31R and R70E) targeted to -6C. B. Data showing significantly improved specificity using this design which was selected as the seed for design extension. C. Using this seed design, five additional residues (Y18, S20, G33, R59, A68) targeted to -6C-5C-4T cluster were selected for randomization to generate a 6C-5C-4T cluster library. D-E. -6C-5C-4T-Ani active variants selected from randomization library had moderate cleavage activity on yeast surface and *in vitro*, that was significantly improved after random mutagenesis and selection. F. Cleavage activity and specificity of -6C-5C-4T-Ani variants (V1 to V3, Supplementary Table S1). All variants showed no activity towards WT and XID (-) full half site. G. Using -6C-5C-4T-Ani variants (V1 to V3) as seed template, I-AniI N-term loop domain (K24, G25, K26, Y27, L28, T29) targeted to the -10A-8T cluster were randomized to extend the design to the full (-) half site. However, this strategy did not generate any active variants (data not shown), suggesting structural shifts caused by multiple mutations at these two regions are not compatible and thus abolish enzyme activity. H. Utilizing an alternative, directed evolution method, XID (-) V1 selected from -6C-5C-4T random mutagenesis library gained cleavage activity towards XID (-) half site (Supplementary Table S1). I. Using XID (-) V1 as template, I-AniI N-term loop domain was re-designed for -10A-8T by randomization. XID (-) V2 selected from N-term loop domain library exhibited increased activity and specificity towards the (-) half site (Supplementary Table S1).

**Supplementary Figure S4.** XID full-site enzyme engineering. A-B. Direct combination of -6C-5C-4T-Ani design and XID (+) half site design generated an -6C-5C-4T+6T+7G+9C+10T-Ani enzyme with moderate cleavage activity on yeast surface, was significantly improved by random mutagenesis and selection as shown on yeast surface and *in vitro*. C. R243W was highly enriched in active variants selected from -6C-5C-4T+6T+7G+9C+10T random mutagenesis library, and this AA change is predicted to affect the positioning of the catalytic domain. D. In the presence of R243W, direct combination of XID (+) and (-) half-site designs generated an active variant towards the XID site (XID-Ani V1, Supplementary Table S1). E. Activity of this variant was further improved by random mutagenesis and selection as shown on yeast surface and *in vitro*.

**Supplementary Figure S5. Specificity of XID-Ani and WT-Ani in TLR assay.** Co-expression of XID-Ani or WT-Ani with TREX2 did not induce NHEJ in 293T reporter cell lines that contained only the WT-Ani or XID target sites, respectively.

**Supplementary Table S1: Ani and XID variants**

| **Ani and XID Variants** | **Mutations** |
|---|---|
| +6T+7G -Ani V1 | Y154A I164R S166I K202Q T204S |
| +6T+7G-Ani V2 | C150S Y154A I164R S166V K202Q |
| +6T+7G-Ani V3 | Y154A I164R S166I K202Q |
| +6T+7G-Ani V4 | Y154A I164R S166V K202N T204S |
| +6T+7G-Ani V5 | Y154A I164R S166V K202Q |
| XID (+) V1 | C150S Y154A L156R N157R D160S Y162S I164R S166I K202Q |
| -6C-5C-4C-Ani V1 | T5K S20G T22S E31R G33V R59S E63K A68R R70E E86D L112Q |
| -6C-5C-4C-Ani V2 | T22S E31R G33V R59I E63K A68K R70E E86D |
| -6C-5C-4C-Ani V3 | T22S E31R G33V R59S E63K A68K R70E E86D |
| XID (- ) V1 | T22S E31R R59I E63K M66T A68K R70E E86D F91L |
| XID (- ) V2 | T22S K24S K26R L28A T29K E31R I55T R59I E63K M66T A68K R70E H76Q E86D F91L |
| -6C-5C-4C+6T+7G+9C+10T -Ani | T22S E31R R59I E63K M66T A68K R70E E86D F91L C150S Y154A L156R N157R D160S Y162S I164R S166I K202Q |
| XID-Ani V1 | T22S K24S K26R L28A T29K E31R R59I E63K M66T A68K R70E E86D F91L L112I C150S Y154A L156R N157R D160S Y162S I164R S166I K202Q K232E R243W |
| XID-Ani | T22S K24S K26R L28A T29K E31R K39R I55T R59I E63K I64T M66T A68K R70E H76Q E86D F91L D122N C150S Y154A L156R N157R D160S Y162S I164R S166I R172K K202Q N226Y K232E R243W |

**Supplementary Table S2: XID homologous sites in mouse genome**

| Mismatches | Chromosome: [start...end] | Strand | XID-Ani tolerability |
|---|---|---|---|
| AGTGCTTGTTTCTCTTGACT<br>     C | **Chromosome 3: [110329864..110329883]** | **-** | partial |
| GGAGCCTGTTTCTCTTGACT<br>A T | **Chromosome 14: [28042251..28042270]** | **+** | complete |
| GGTGCCTGTTTCTCTTGCCT<br>A                A | **Chromosome 4:[89679652..89679671]** | **-** | complete |
| TGTGCCTGTTTCTCCTGACT<br>A             T | **Chromosome X: [86536949..86536968]** | **-** | complete |
| TGTGCCTGTTTCTCTTGACA<br>A                  T | **Chromosome 19:[59991764..59991783]** | **+** | no |
| AATGCCTGTTCCTCTTGACT<br> G        T | **Chromosome 11: [7458936..7458955]** | **+** | no |
| AATGCCTGTTTCTCTTCACT<br> G              G | **Chromosome 17: [93812304..93812323]** | **+** | **no** |
| ACTTCCTGTTTCTCTTGACT<br> G G | **Chromosome 10:[69720061..69720080]** | **-** | no |
| ACTGCCTGTTTCCCTTGACT<br> G          T | **Chromosome X:[45845778..45845797]** | **+** | no |
| ATTGCCTGTTTTTCTTGACT<br> G         C | **Chromosome 9:[88346296..88346315]** | **+** | no |
| AGCGCCTGTTTCTCTTGACT<br>  T | **Chromosome X: [131108498..131108517]** | **-** | BTK WT partial |
| AGTTCCTGTTGCTCTTGACT<br>   G      T | **Chromosome 16: [21773580..21773599]** | **-** | no |

| | | | |
|---|---|---|---|
| AGTTCCTGTTTCTCATGACT<br>G T | **Chromosome 14: [100159753..100159772]** | **+** | no |
| AGTGACTGTTTCTCCTGACT<br>C T | **Chromosome 17: [66337098..66337117]** | **-** | complete |
| AGTGGCTGTTTCTCTTGCCT<br>C A | **Chromosome X: [39701370..39701389]** | **+** | complete |
| AGTGTCTGTTTCTCTAGACT<br>C T | **Chromosome 9: [35256345..35256364]** | **+** | no |
| AGTGTCTGTTTCTCTTTACT<br>C G | **Chromosome 9: [90510956..90510975]** | **+** | no |
| AGTGCCTGCTACTCTTGACT<br>T T | **Chromosome 2: [142108391..142108410]** | **+** | no |
| AGTGCCTGTTAATCTTGACT<br>TC | **Chromosome 11: [78504298..78504317]** | **+** | no |
| AGTGCCTGTTACTCTTGACC<br>T T | **Chromosome 12:[89556487..89556506]** | **-** | complete |
| AGTGCCTGTTTCTTTTGGCT<br>C A | **Chromosome 15: [49534970..49534989]** | **-** | no |
| AGTGCCTGTTTCTCCTGATT<br>T C | **Chromosome 4: [50511967..50511986]** | **+** | complete |
| AGTGCCTGTTTCTCTTTACA<br>G T | **Chromosome X: [135770550..135770569]** | **-** | no |

**Supplementary Table S3: primers for genomic target site amplification**

| Primer | Sequence |
|---|---|
| XID FP | CACCATGTAGAATCAATCCCCTGG |
| XID RP | CCCCTGCCATGACTGTGCCTAC |
| -10G-8A FP | ATTGCTCCTGGCTTCTTCAG |
| -10G-8A RP | CTTTGAACCTGGTACTCCAG |
| -6A+5C FP | ATCTTACACATATCAGCCAG |
| -6A+5C RP | TTTGGCTGACTCTAACCAAC |
| +5C+9T FP | AAGTGCAAGTTGAGAGCAAC |
| +5C+9T RP | TTTGGTCAGGGCCTGATAGC |
| -5T FP | GCATGCATTTCAATATCGAC |
| -5T RP | AGAATCCCTTTGTTTGTACC |
| +10T-10A FP | GGTGGTACCAAATTCTATCC |
| +10T-10A RP | ATTTTGGGATGCCCTACCTC |
| -2C+1A FP | TAGTACCATTTGGGTGATCC |
| -2C+1A RP | AGGTTAGGCAGTGAAGGAGC |
| +4T+8G FP | TCTGAGCCTTTAAGATTCTG |
| +4T+8G RP | CATACTGTATATTCTCACTGTG |
| TLR HDR FP | AAGGGCGAGGAGCTGTTCACC |
| TLR HDR RP | AGCTCGTCCATGCCGAGAGTGATCC |
| TLR NHEJ FP | AAGGGCGAGGAGCTGTTCACC |
| TLR NHEJ RP | CTGCCGTCCTCGATGTTGTG |

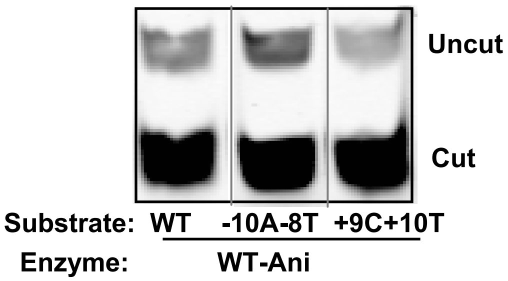


# Supplementary Figure S1

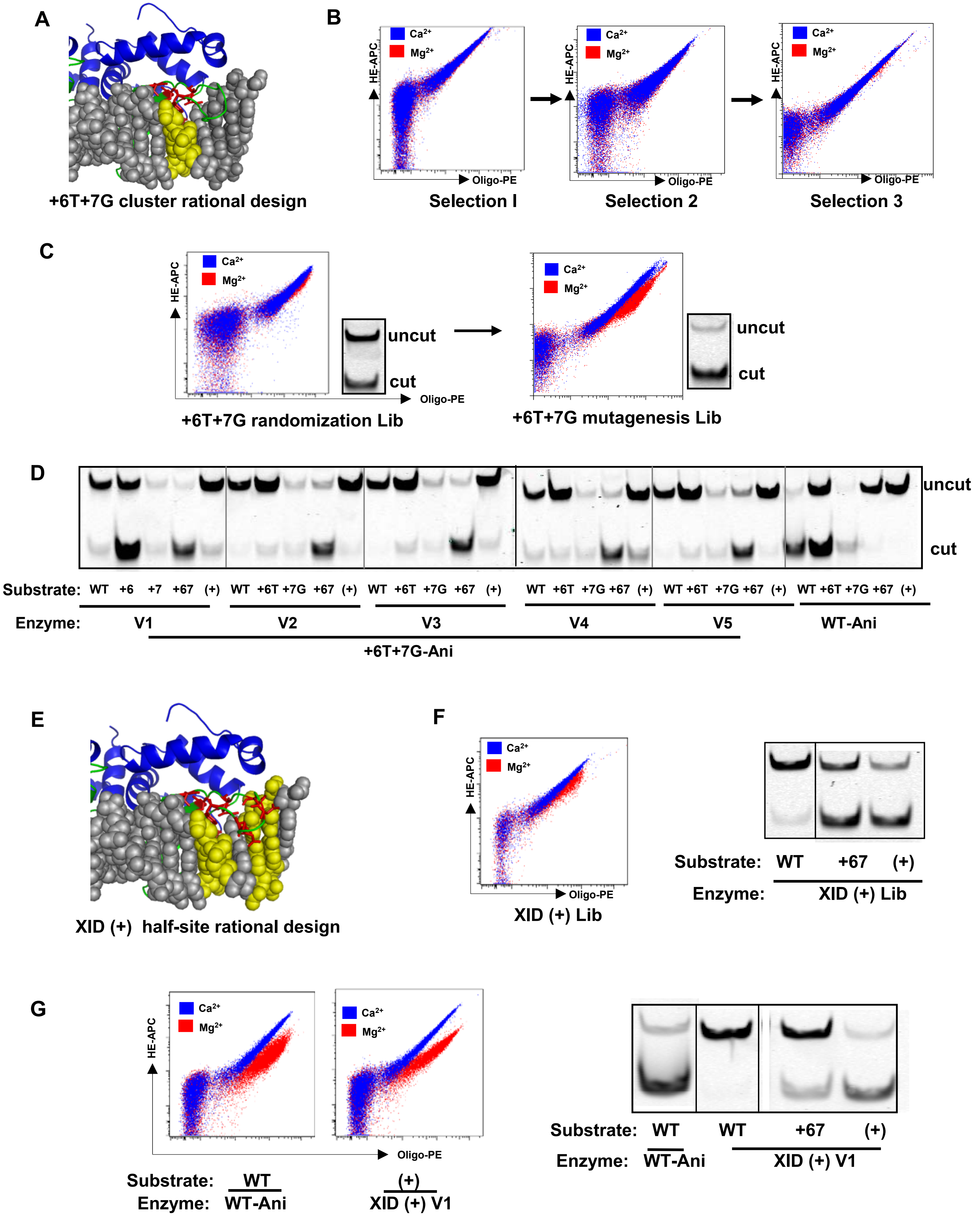


# Supplementary Figure S2

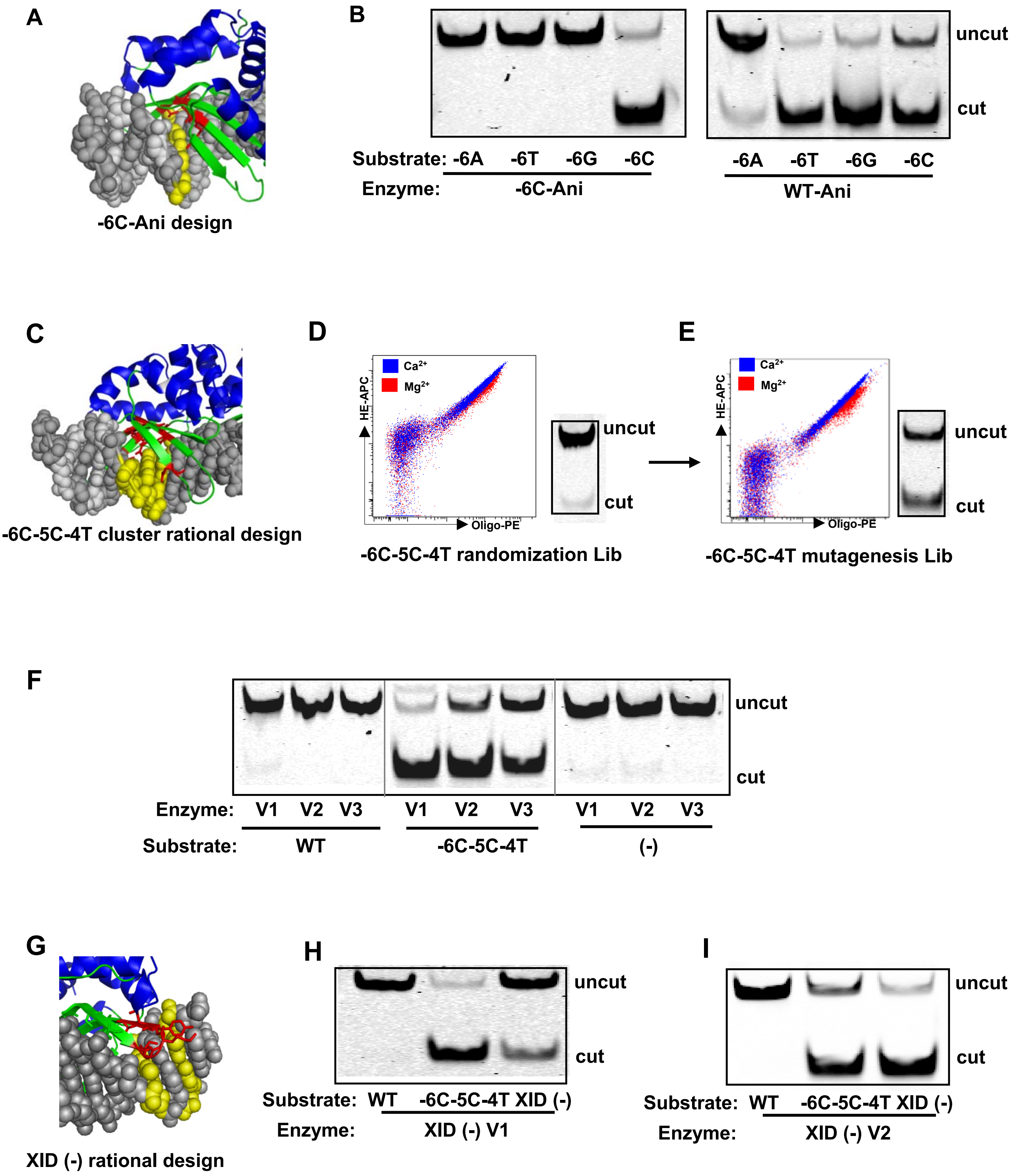


# Supplementary Figure S3

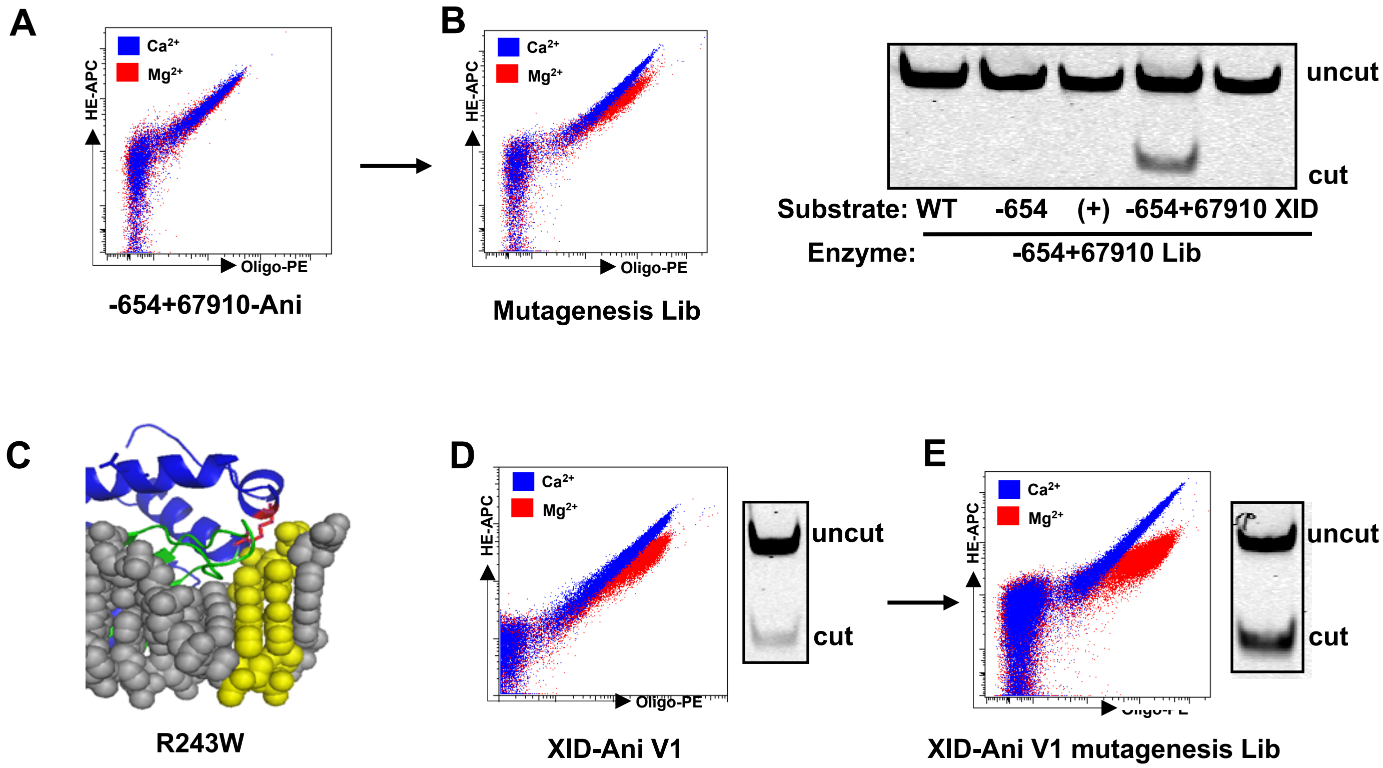


# Supplementary Figure S4

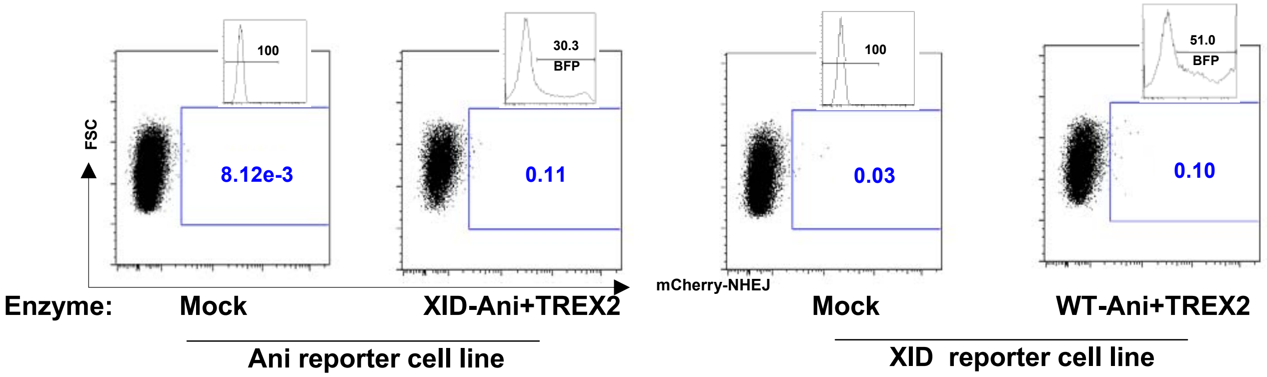


# Supplementary Figure S5
